# Student apathy for classroom learning and need of repositioning in present andragogy in Indian dental schools

**DOI:** 10.1186/1472-6920-12-118

**Published:** 2012-11-24

**Authors:** Rajani A Dable, Babita R Pawar, Jaykumar R Gade, Prasanth M Anandan, Girish S Nazirkar, Jyoti T Karani

**Affiliations:** 1 Department of Prosthodontics, SMBT Dental College and Hospital and Research Institute, Ghulewadi (Amrutnagar) P.O., Ahmednagar, 422608, Maharashtra, India; 2 Department of Periodontics, Pravara Rural Dental College & Hospital, Loni, Maharashtra, India; 3 Department of Prosthodontics, SDKS Dental college and hospital, Wana Dongri, Nagpur, Maharashtra, India; 4 Department of Pedodontics, Vyas Dental College & Hospital, Kudi Haud, Jodhpur, Rajasthan, India; 5 Department of Prosthodontics, SMBT Dental College and Hospital and Research Institute, Ghulewadi (Amrutnagar) P.O., Sangamner, Maharashtra, India; 6 Department of Prosthodontics, Terna Dental College & Hospital, Nerul (W), Navi Mumbai, Navi Mumbai, Maharashtra, India

**Keywords:** Contemporary education, Student psychology, Teacher development, Classroom teaching, Distance learning, Andragogy, Dental education

## Abstract

**Background:**

In the world of technology, when today's student is approaching the on-line /distance learning in the open universities and doing on-line self-assessment, the classroom learning is vanishing slowly. Globally, teachers are taking efforts to improve the pedagogy by implementing effective methods to retain the classroom teaching and student attendance. The present study aims at shedding some light on the need of changing the adult education strategies (andragogy), which can effectively improve the student attendance for lectures.

**Methods:**

It is an observational study, and the conceptual framework of it is based on beliefs, opinions and personal experiences of the respondents. Triangulation method is used for collecting the data. The data is achieved from three groups of concerned population who could provide valid results to support the study. It is collected by interviewing 10 senior faculty members who are/were the 'education experts' in the universities, while the main concerned groups of present educational stream, i.e. 'institution-teachers' and the 'students', were given questionnaires. 570 teacher respondents and 200 student respondents are the main participants of this study.

**Results:**

As per data, it has been observed that senior faculty (90%) and students (93.25%) feel need of student motivation more than the institutional teachers (52.44%). P-values were obtained using Chi-Square test for testing the significance of difference between agreement and disagreement for a specific question.

**Conclusions:**

In India, Universities have already sensed the need of 'teacher development programmes'. But teachers in dental colleges, demand more efforts to be taken by universities and managements in this regard and expect better educational policies to give them accessibility to prove themselves.

## Background

Teaching has become the most challenging profession today with increasing demands and expectations by students. Unfavorable conditions, such as increasing class size and decreasing classroom-learning interest among pupils, has made it a crucial issue. Student absenteeism is a common problem and does exist in dental colleges as well. Bertolami states that “one of the biggest tip-offs that the form of dental education needs revision is the simple observation that dental students do not, in general, like dental school” [1]. Understanding some of the concerns of dental students might help faculty and administrators modify or change existing programs to meet some of the areas that have been identified as deficient when data from the DSLES (Dental Student Learning Environment Survey) was analyzed [2].This shows the need of improving the educational strategies to higher level.

The teachers need to understand and improve their andragogical (teaching methodology for adult learners) methods to suit the present needs. The international literature shows considerable interest in student absenteeism: its effects and implications for the individual learner, for university lecturers, and for institutions [3]. Students need to be convinced about the benefits of attending lectures. At the same time it is important to know the reason behind this apathy. Today the educational strategies are changing, now it is possible to transmit educational courses, programs and content widely using the various mass media distribution channels [4]. The development of the world-wide-web and satellite enables even broader access to university courses.

A study by Kent provided the results which present a considerable challenge for psychologists and sociologists teaching at dental schools and suggest that appropriate curriculum design and integrated teaching may be the way forward [5].

### Role of teacher as motivator

Andragogy defines adult education which deals with adult learners. Adult learner is always a self-directed learner who understands his/her own responsibility. Motivation of these learners is a vital factor in institutions, where teachers have to adapt specific tools to change the students’ perspective of looking at things. As dentistry necessitates more of technical skills, students should be induced by teachers to involve themselves more in artistic work, eg. making dental models, charts, concept maps, collages etc. which can draw them to the classroom. In professional programs like medicine, dentistry, dental hygiene, and nursing, effective teachers are produced by happenstance rather than design. The expert knowledge and technical skills of professionals are assumed to “serve as adequate qualifications” for effective teaching, although this is an “antiquated theory” [6].

To make the teaching effective, it is necessary for the present day teacher to be aware of the changes that are taking place in health education. The changes are: shift from conventional role of teacher, changes in learning styles, innovative curriculum models and changes in assessment philosophy, methods and tools [7].

Although the literature on effective teaching in dentistry and dental hygiene provides information for the classroom, laboratory, and clinic, it is limited, requiring much more research attention [8].

There are gaps in studies on dentistry which need to be filled by understanding its extensive need. Studies on effective teaching behaviors in the classroom are limited in dentistry and dental hygiene, and reveal that effective teaching in the classroom includes behaviors such as organization, enthusiasm, empathy, rapport, clarity, general knowledge, and being available for students [9].

Measuring teaching effectiveness is also important to make required changes in academics.

There are national standards for how teaching effectiveness or performance should be measured—the *Standards for Educational and Psychological Testing* (AERA, APA, & NCME Joint Committee on Standards, 1999) [10].

Classroom communication systems (CCS) are technology products designed to promote communication and interactivity in big classes making them small. In an economic context, where universities are facing rising competition with the distance education and self-paced learning programmes, they must improve the educational strategies to keep the students and teachers face-to-face [11].

### Faculty development

Teachers are accustomed to teaching students, but experienced teachers must also teach teachers; and effective teachers come with a variety of styles and personalities [12]. ‘Health Survey and Development Committee’ recognized the need for training of medical teachers as early as in 1946 and made recommendations. Nearly three decades later, efforts towards this began.

An expert committee of the World Health Organization (WHO) in 1965 brought out a report on, “The training of teachers of medical schools with special regard to developing countries”. In India the first ‘National Course on Teacher Training’ was held in March 1976, with support from WHO. For faculty development, the factors like fellowships and travel grants, centers of health professional education, recognition and rewards to the efficient academics, should be taken into consideration to encourage specialization in education [13].

The universities and the institutions must encourage the teachers to realize the necessity of updating their teaching. And teachers have to take efforts to retain the ‘classroom teaching’ as the technology is taking the adult education out of classroom making it more universal online. The distance education technologies are expanding at an extremely rapid rate [14].

The aim of this study is to explore the attitude of teachers towards motivating students for classroom learning and their perspective towards the need of changing and developing the present andragogical methods. This has been achieved by comparing the views of students and teachers; that can give a clear idea about the present ‘educational need’ of the students in dental colleges. At the same time the study reveals the factors which hinder teacher development.

## Methods

It is an investigative, observational, cross sectional survey study. The survey was designed first to determine the population for the study and the questionnaire was formatted according to it. The study framework was based on the beliefs, opinions and personal experiences of the respondents. The data were derived by a triangulation method where the respondents were taken from three different groups of population which helped in increasing the validity of evaluation and research findings.

Three different ways were chosen as the survey method, namely; email, telephone and personal interviewing. The initial planning of this study was done at SMBTDC (Sau Mathurabai Bhausaheb Thorat Dental College) Sangamner. To make the study more generalized, seven colleges were sent the invitation to make their students to participate out of which only four of them could take part as per our convenience in the stipulated time period. Fifty students from each college were selected randomly to participate (Table 1). The teachers from different colleges of India participated in this study. An ethical approval has been obtained for this study from the SMBT Dental College Ethical Board.

**Table 1:** College wise rating of students’ perception

**Questions**	**SMBTDC (n=50)**	**PRDC (n=50)**	**SDKSDC (n=50)**	**VDC (n=50)**	**All (n=200)**	**P-value**
Do you enjoy present ‘classroom teaching/learning?	21(42.0%)	19 (38.0%)	22 (44.0%)	24(48.0%)	86 (43.0%)	0.048
Will you prefer classroom learning over distance learning?	34 (68.0%)	35 (70.0%)	35 (70.0%)	33 (66.0%)	137 (68.5%)	0.001
Are the lectures too lengthy and ineffective to understand?	35 (70.0%)	33 (66.0%)	37 (74.0%)	32 (64.0%)	137 (68.5%)	0.001
Is that the main reason to remain absent for classes?	34 (68.0%)	40 (80.0%)	35 (70.0%)	37 (74.0%)	146 (73.0%)	0.001
Do you attend the classes just for assessments and feedbacks?	12 (24.0%)	10 (20.0%)	10 (20.0%)	6 (12.0%)	38 (19.0%)	0.001
Do you think teacher motivation can improve ‘student- attendance’ for lectures?	49 (98.0%)	47 (94.0%)	46 (92.0%)	45 (90.0%)	187 (93.5%)	0.001
Do you think pedagogical methods need to be changed to benefit the students?	39 (78.0%)	44 (88.0%)	49 (98.0%)	44 (88.0%)	176 (88.0%)	0.001
Do you think better pedagogical methods can improve ‘classroom learning.’	45 (90.0%)	45 (90.0%)	47 (94.0%)	44 (88.0%)	181 (90.5%)	0.001

(Values are n (% of respondents) who mean ‘Yes’ for the respective question).

SMBTDC- Sau Mathurabai BhausahebThorat Dental College.

PRDC- Pravara Rural Dental College.

SDKSDC- Swargiya Dadasaheb Kalmegh Smruti Dental College.

VDC- Vyas Dental College.

The time period scheduled for the study was two months (61 days), which determined the sample size of the study. The quantitative data was derived from two main groups, the institutional teachers and the students. The qualitative data was derived from the senior faculty which was taken to compliment the data derived from the other two groups.

### Interviews (senior faculty)

The interviews of ten senior teachers from different institutions and the universities were taken. The criteria for selection was their vast knowledge and dedication in the field of dentistry as academician. They were randomly selected from the age group 55–62. Following were the questions asked in the interviews. Some of these interviews were taken face to face while a few on telephone. The specific interview questions included:

1. *Do you think teacher motivation and better pedagogical methods can improve ‘classroom teaching’ and ‘student- attendance’ for lectures?*

2. *Do you think teachers need to change the present pedagogical methods and make them more effective to match the present time?*

3. *Do you think classroom teaching is a better pedagogical method than online/ distance learning?*

4. *In your view what are the other factors responsible for student absenteeism?*

### Questionnaire for students

Totally 200 students participated in this study. To receive the comparative data, 50 students were selected randomly from IV year BDS course (the most experienced students in college) from four dental colleges in the states of Maharashtra and Rajasthan. A structured questionnaire comprising of 7 questions (about the standard of today’s pedagogy and need of teachers’ motivation in classroom teaching), based on ‘Yes’ and ‘No’ scale was given to students.

### Questionnaire for teachers

The questionnaires were distributed to 700 teachers out of which total 570 were collected in a stipulated time period of 61 days. Test pilot survey was done for 10 teachers and students to evaluate the competency of the questionnaire followed by circulation among 700 participants. The ‘Target Population’ comprised those teachers who were actively involved in teaching clinical as well as nonclinical subjects. Simple random sampling technique was used to collect the information. The response rate for the study was 81.43%.

The respondents were asked to answer 7 questions regarding the strength of their agreement based on five Likert scale ranging from ‘strongly agree’ to ‘strongly disagree.

### Statistical analysis

Data is shown as n (% of respondents) for each group of respondents. In order to compare the combined responses of students from all four colleges Chi-square test is used with a null hypothesis that ‘Yes’ and ‘No’ responses are likely to occur equally (Table 1). The teacher’s perspective has been categorized to three categories by combing the disagree/agree and strongly disagree/agree responses together for the sake of simplicity. Here p-values are obtained using Chi-Square test for testing the significance of difference between agreement and disagreement perspective for a specific question (Table 2). Finally the perspectives of all three groups of respondents have been compared using Chi-Square test if cell frequencies are greater than 5, else Fisher’s exact probability test is applied for this purpose (Tables 3 and 4). The p-values less than 0.05 were considered statistically significant. All the hypotheses were formulated using two tailed alternatives against each null hypothesis. The entire data was statistically analyzed using ‘Statistical Package for Social Sciences’ (SPSS ver 11.5, Inc. Chicago, USA) for MS Windows.

**Table 2:** Perception of teachers

**QUESTIONS**	**Total respondents (n=570)**			**P-value**
	**Disagree/Strongly disagree**	**Agree/Strongly agree**	**Neither agree nor disagree**	**(Agree v/s Disagree)**
1. Teacher motivation can improve ‘student- attendance’ for lectures	116 (20.4)	**299** (**52**.**5**)	155 (27.1)	0.001
2. Better pedagogical methods can improve ‘classroom teaching’	31 (5.4)	**502** (**88**.**1**)	37 (6.5)	0.001
3. Teachers need to change the present pedagogical methods and make them more effective to match the present time.	59 (10.4)	**477** (**83**.**7**)	34 (5.9)	0.001
4. Classroom teaching is the better pedagogical method than online/distance learning	105 (18.4)	**415** (**72**.**8**)	50 (8.8)	0.001
5. Teachers’ support in learning can motivate students.	129 (22.6)	**325** (**57**.**0**)	116 (20.4)	0.001
6. Assessments & feedbacks make students attend the classes.	138 (24.2)	**410** (**71**.**9**)	22 (3.9)	0.001
7. Teachers’ should get incentives for their academic achievements in the form of free trainings, free academic trips abroad, money etc.	74 (12.9)	**437** (**76**.**7**)	59 (10.4)	0.001
8. The present education policies are satisfactory.	**345** (**60**.**5**)	102 (17.9)	123 (21.6)	0.001
9. There should be a Centre for Professional Development (CPD) for academicians in every institution.	181 (31.8)	**328** (**57**.**5**)	61 (10.7)	0.001

(Values are n (% of respondents). Significantly higher proportion of responses has been shown in bold text).

**Table 3:** Comparative data between three groups of respondents

**QUESTIONS**	**Senior faculty (n=10)**	**Institutional Teachers (n=570)**	**Students (n=200)**
1. Teacher motivation can improve ‘student- attendance’ for lectures	9 (90.0%)	299 (52.4%)	187 (93.3%)
2. Better pedagogical methods can improve ‘classroom teaching’	9 (90.0%)	502 (88.1%)	181 (90.3%)
3. Teachers need to change the present pedagogical methods & make them more effective.	9 (90.0%)	477 (83.7%)	175 (87.3%)
4. Classroom teaching is the best pedagogical method than online/distance learning	8 (80.0%)	415 (72.8%)	135 (67.5%)

Values are n (% of respondents).

**Table 4:** **Comparative data between three groups of respondents** (**Statistical comparison**)

**QUESTIONS**	**Senior faculty v/s Institutional teachers**	**Senior faculty v/s Students**	**Institutional teachers v/s Students**
1. Teacher motivation can improve ‘student- attendance’ for lectures	0.023	0.506	0.001
2. Better pedagogical methods can improve ‘classroom teaching’	0.999	0.999	0.350
3. Teachers need to change the present pedagogical methods & make them more effective.	0.999	0.999	0.197
4. Classroom teaching is the best pedagogical method than online/ distance learning	0.999	0.508	0.153

Values are p-values, obtained by chi-square test P-value<0.05 is considered to be statistically significant.

## Results

### Students’ perception

Table 1 shows the overall and college specific perception of students on various issues regarding conventional class room learning. The students expected their teachers to come up with novel and interesting methods of teaching. Since significantly higher proportion of students (57%) responded that they did not enjoy present ‘classroom learning (p<0.05). This reflected apathy of students for the present classroom learning, which could be the main reason for the absenteeism. Also, a significantly higher proportion of students responded that they preferred class room learning to online/ distance learning (p<0.001). When asked if they would prefer classroom learning over online/distance learning or any other method, 68.5% expressed agreement. Also a similar proportion (68.5%) agreed that the lengthy and ineffective lectures proved to be tiring, increased their boredom, making them stay away from the classes. Further, 73.0% students agreed that it was the main reason to remain absent for the classes. Only 19.0% of the total said that they attended the classes just for assessments and feedbacks while 81.0% attended the classes because they needed the teachers’ support in learning. A total of 93.5% students thought that teacher motivation could improve ‘student attendance’ for lectures, 88.0% students thought that today’s pedagogical methods needed to be changed for students’ benefits and 90.5% students thought that better pedagogical methods could improve ‘classroom learning (p<0.001 for all questions except a question on enjoying classroom teaching) (Table 1).

### Teachers’ perception

Table 2 depicts the perception of students on various issues regarding conventional class room learning. It is clear that significantly higher proportion of teachers agreed that their motivation could improve the student’s attendance for lectures (p<0.001). A significantly higher proportion of teachers agreed better pedagogical methods could improve classroom teaching (p<0.001). P-values were obtained using Chi-Square test for testing the significance of difference between agreement and disagreement for a specific question.

The results showed that, 52.5% teachers agreed that proper motivation could improve ‘student- attendance’ for lectures. A total of 88.1% teachers admitted that better pedagogical methods could improve the classroom teaching and make it more interesting for students.

Majority of teachers (83.7%) agreed to the fact that teachers needed to change the present pedagogical methods and make them more effective to match the present times while, 10.4% of them disagreed with this as they thought that the basic pedagogical methods and effective communication were more important factors. Also, 72.8% respondents felt that classroom teaching was a better pedagogical method than online/distance learning as classroom teaching was a face to face teaching which could make the students understand the subject and clear the doubts by efficient guidance of an experienced teacher. The rest 18.4% thought that online or distance learning was more beneficial for students as they could anytime learn as per their convenience and it could be more informative than classroom learning.

About 57.0% teachers agreed that students needed the full support of teachers in solving their doubts. They also stated that students were very much interested in knowing the feedback and remarks about their assessments while 22.6% teachers thought that students didn’t really need teachers' support as they believed in self study or took senior students' help to clear their doubts. Approximately 71.9% teachers noticed that assessments & feedbacks with encouraging approach made students attend classes. It was seen that 76.7% teachers thought that the universities and the institutions should provide them with incentives in some form for their academic achievements, in the form of free trainings, free academic trips abroad, money etc. so that they got encouraged to perform better. At the same time 12.9% teachers felt that teachers did not need incentives; they were professionally qualified people and should know how to make their job the best even without any incentives. 60.5% teachers were not very happy with the present education policies as they thought they should be changed with improved educational standards in favor of teachers as well as students, which could definitely improve the whole scenario. Only 17.9% of them felt that the present policies were satisfactory. Finally, 57.5 % respondents felt that there should be a centre for professional development (CPD) for academicians in every institution, which could arrange teacher training programmes to update their knowledge about the current pedagogical strategies, while 31.8% still did not feel any need of it (p<0.001 for all questions) (Table 2).

### Senior faculty interviews

From the interviews conducted, it was rightly concluded that 9 (90%) senior faculty members felt that teacher motivation and better pedagogical methods could improve ‘classroom teaching’ and ‘student- attendance’ for lectures, while only 1 (10%) faculty thought that effective presentation and communication could lure the students to the classroom.

When the senior faculty was asked whether the teachers needed to change the present pedagogical methods and make them more effective to match the present time, 9 (90%) of them strongly supported it, though 2 (20%) of them favored the need of effective basic pedagogy, and only 1 (10%) member thought it was not required. Eight members (80%) thought that classroom teaching was a better pedagogical method than online/ distance learning as students were more benefitted in the presence of the teacher as a guide, while 2 (20%) members thought that on-line/distance learning could also be an ideal source that students could find beneficial in the present times.

There are various factors responsible for student absenteeism, but the senior faculty felt that teachers, students, institution management, all were responsible for this scenario. Whereas 30 % of them thought that students were more responsible as they should know their responsibility, 40% of them thought responsibility was to be taken up by teachers, if they wanted to change the scenario. The rest, 30%, felt that parents and the institutional management should be more strict as far as absenteeism is concerned.

### Comparison of perspectives of all three groups

Comparative analysis was done between three groups of respondents with different category leading to confirmation of argument through divergence with dissimilar conclusions in some areas, while the severity of perception and need was placed differently, but the intention was similar in other areas (Table 3).

**Figure 1:**
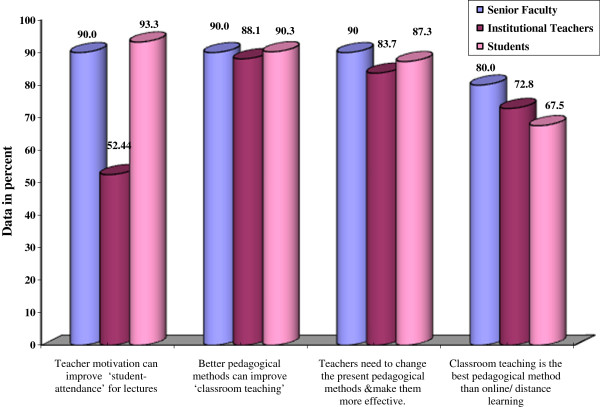
Comparative data of all three groups.

Senior faculty and institutional teachers did not significantly agree with “teacher motivation can improve ‘student- attendance’ for lectures” (p<0.05). Institutional teachers and students did not significantly agree with “teacher motivation can improve ‘student- attendance’ for lectures” (p<0.001). The three groups of respondents agreed with each other on all other questions (Table 4). Senior faculty, as per their substantial experience and knowledge, expressed their support for the need of student motivation by teachers as well as the need of change of andragogical methods (Figure 1).

## Discussion

Both in the United States and abroad, undergraduates have begun to view themselves as “learning consumers” with expectations to be met in their education [14]. Student absenteeism, motivation, teacher’ support, teachers’ development; all these factors are interconnected. One cannot be achieved without the other. And this is what the need of today’s teaching faculty is; to understand its connectivity.

In one of her articles, Carole A. Ames (University of Illinois), clarifies the complex construct of motivation as it relates to learning, and offers revamped curriculum that applies motivation theory and research to practice.

As per Terrell H. Bell there are three things to remember about education. The first one is motivation. The second one is motivation. The third one is motivation. It shows that, literature has stressed on motivation in education as a very important aspect of it.

Student assessment is also an important factor. Application of more effective and innovative assessment methods can change the student learning. If we find our systems do not allow us to implement a really valuable assessment innovation, for example, then we must find ways to change the system [15]. As per Brown, “if you want to change student learning, then change the method of assessment”, [16]. When the evidence is actually used to adapt the teaching work to meet learning need is called ‘formative’, according to Anderson, it promotes skills transfer and deeper level understandings [17]. There is a new wave of pedagogy advocating ‘authentic assessment’ that is based on the *constructivist* approach , enabling students to demonstrate knowledge by working on authentic tasks [18].

Classroom teaching is still a preferred method of education though distance /online/mobile education methods are coming up. Various studies have been done on distance education vs traditional classroom education with/without significant data. One such study by Emily Mirakian concluded that although the online students were highly satisfied with the course and their self-perceived knowledge gains, the online satisfaction ratings were generally lower than those found in the traditional courses [19] This study supports our study where the students are in favor of online learning but prefer the traditional (classroom) learning.

A study by Emeka Nkenke et al. compared the opinions of III year dental students between the traditional group and the technology-enhanced learning group. Both groups were positive about the flexibility that e-learning can give as far as time and place of learning are concerned. However, the answers of both groups indicated that face-to-face lectures were still considered the basis for learning at university and that the lecturer had a strong influence on the students’ interest in a specific subject [20]. This study supports the present study where the students think that the teachers have strong influence on students as they can motivate them the right way.

Though it is a controversial issue, many researchers found no significant difference in satisfaction, motivation or achievement between online and traditional learners [21].Other investigators found that online learning can be as effective as traditional learning [22]. Harris and Parrish (2006) found that when they compared two courses, one online and one traditional, that there was a significant difference in the learning outcomes between two courses and that the in-class students received significantly higher grades and had a lower dropout rate [23]. On the contrary, according to a 2009 study conducted by the U.S. Department of Education, which reviewed more than 1,000 studies conducted on online learning between 1996 and 2008, students performed better in an online education situation than in face to-face situations, on average [24].

Thus the opinions about the education methodologies are in contrast to each other and biased. As per the present study, though the students are not in favour of the present classroom teaching, they do expect a better classroom environment with better/ updated teaching.

‘Teacher development’ is an important factor, and the teacher needs to take it as a challenge to keep himself the best source of knowledge as far as the classroom teaching is concerned. To achieve the innovative methods and learn their implications, teachers need to undergo various developmental programs. The Indian universities especially Maharashtra University (MUHS) has undertaken many teacher development programmes for updating teachers in syllabi pattern, examination reforms, professional knowledge and communication skills. So far, 64 workshops for teachers have been conducted in which nearly 4600 teachers of all faculties have been trained [25].

## Conclusions

The main issue raised and discussed throughout is that the need of effective teaching requires the teachers to utilize a range of teaching and assessment approaches and methods in order to enhance learning. Distance learning and all other advanced and contemporary educational methodologies are trying to replace classroom teaching. It is reported in this study that the learners are demanding better classroom teaching which can draw them towards it. It is important for an institutional teacher to explore the current adult learning theory, and relate it to the current practice of teaching. Variety of factors that influence teaching and learning include social and individual psychological aspects of adult learning, patterns of participation, student motivation, classroom behaviors, and assessment and evaluation strategies.

As per this study, the senior faculty and students feel a more intense need of student motivation as well as implementation of better pedagogical methods than the institutional teachers. It shows that the institutional teachers need to change their perception with inclination for change as needed with time. Senior faculty and the institutional teachers are more in favor of classroom teaching and consider it a better pedagogical method than online/distance learning. On the contrary, the students do not find it that beneficial.

In India, though the universities have sensed the need of ‘teacher development programmes’, teachers have appealed to the universities and the managements for more practically effective policies which can lead to their ‘self growth’ as a ‘teacher’ which can certainly help them change the whole scenario.

## Competing interests

The authors has/have academic and intellectual competing interest.

## Authors’ contributions

RD has played a major role in conception and designing of the study, acquisition, analysis and interpretation of data, also finalizing the manuscript. JG has participated in the acquisition of study material, and data collection from various parts of country. BP helped in drafting the manuscript and scrutinizing the data. PMA supervised the data collection staff, and participated in the sequence alignment and design of the study. GN and JK helped in drafting and finalizing the manuscript. All authors read and approved the final manuscript.

## Pre-publication history

The pre-publication history for this paper can be accessed here:

http://www.biomedcentral.com/1472-6920/12/118/prepub
